# Chinese Herbal Medicine (Chaihu-Huaji Decoction) Alleviates Postembolization Syndrome following Transcatheter Arterial Chemoembolization and Improves Survival in Unresectable Hepatocellular Cancer: A Retrospective Study

**DOI:** 10.1155/2019/6269518

**Published:** 2019-02-03

**Authors:** Hua Xu, Yongchun Deng, Zhi Zhou, Yi Huang

**Affiliations:** ^1^Chongqing Hospital of Traditional Chinese Medicine, No. 6, 7 Branch Road, Panxi, Jiangbei District, 400021 Chongqing, China; ^2^Chongqing Key Laboratory of Translational Research for Cancer Metastasis and Individualized Treatment, Chongqing University Cancer Hospital & Chongqing Cancer Institute & Chongqing Cancer Hospital, No. 181, Hanyu Road, Shapingba District, 400030 Chongqing, China; ^3^Department of Infectious Diseases, Institute for Viral Hepatitis, Key Laboratory of Molecular Biology for Infectious Diseases, Ministry of Education, Second Affiliated Hospital of Chongqing Medical University, No. 76, Linjiang Road, Yuzhong District, 400010 Chongqing, China; ^4^Chongqing Hospital of Traditional Chinese Medicine, Forth School of Clinic Medicine of Chendu University T.C.M., No. 6, 7 Branch Road, Panxi, Jiangbei District, 400021 Chongqing, China

## Abstract

**Objective:**

To examine the efficacy of Chaihu-huaji decoction combined with transarterial chemoembolization (TACE) in unresectable hepatocellular carcinoma (HCC) patients.

**Methods:**

We retrospectively reviewed the data of 125 HCC patients treated in Chongqing Cancer Hospital between January 2012 and December 2014, including 64 patients who received Chaihu-huaji decoction and TACE (Chaihu-huaji group) and 61 patients who received only TACE (control group). The patients were examined until the last follow-up or death. Complications, hepatotoxicity, and nephrotoxicity were compared between the two groups.

**Results:**

Fever, nausea, vomiting, poor appetite, and leukocytopenia were alleviated in patients who received Chaihu-huaji decoction, and no cases of significant hepatic and renal toxicities related to the herbal medicine were observed in the Chaihu-huaji group. The 3-year overall survival probability was significantly higher in the Chaihu-huaji group (26.47%) than in the control group (13.06%).

**Conclusion:**

Chaihu-huaji decoction may prevent adverse events after TACE and prolong overall survival of unresectable HCC patients when combined with TACE.

## 1. Introduction

According to GLOBOCAN 2012 [1], liver cancer is the sixth most common type of cancer and the second leading cause of cancer-related death, with an estimated 782,000 new liver cancer cases occurring worldwide and 746,000 people having died of this disease in 2012 (50% of them in China alone). According to the 2015 Cancer Statistics of China [2], the liver cancer incidence and mortality were 466,100 and 422,100, respectively, from 2009 to 2011, making it the fourth most common cancer and the third leading cause of death in China. Hepatocellular carcinoma (HCC) is the main type of liver cancer.

Liver resection and liver transplantation are curative treatments for early-stage HCC [3–5]. However, most HCC patients are diagnosed at intermediate to advanced stages, and for these patients, several treatment options may be considered. These consist most commonly of repeated hepatectomy (RH), radiofrequency ablation (RFA), and transarterial chemoembolization (TACE) [6–8]. For tumors of greater size and number in the liver, TACE would be more appropriate [9]; however, postembolization syndrome (PES) following TACE, which is characterized by a series of symptoms, including fever, nausea, vomiting, Poor appetite, abdominal pain, and liver function impairment, can lead to a reduction in the quality of life (QOL) of patients, even prompting withdrawal from treatment.

The use of complementary and alternative medicine (CAM) among cancer patients in western countries has been increasing in recent years [10]. Traditional Chinese herbal medicine (CHM), which has been widely used in China and east Asia as a main CAM therapy, has already become a commonly used treatment for cancer [11]. And, recent studies indicate that TCM could play an important role in the whole course of cancer prevention and treatment, including prevention of tumorigenesis, reducing tumor recurrence and metastasis, and alleviating and even eliminating the toxic effects of the treatments, controlling symptoms [12–14], improving QOL, prognosis, and overall efficacy [15–18]. In the treatment of liver cancer, CHM is effective in prolonging life expectancy, improving immune response, and reducing side effects of TACE [19]. For example, the FuZheng GuBen decoction can promote the recovery of liver physiological function [20], and Jianpi Ligan decoction (JLD) can improve the efficacy and relieve PES following TACE in unresectable primary liver cancer [21, 22].

Small bupleurum decoction (Xiaochaihu-tang in Chinese; and Sho-saiko-to in Japanese) can strengthen the body's resistance to pathogenic factors and has long been used in the treatment of chronic liver diseases such as hepatitis and liver cirrhosis in China, Korea, and Japan [23]. It is also useful in preventing inflammation and cancer progression [24–26] and can be used in treating cancer cachexia [27]. Abdominal mass pill (Huayu Pill in Chinese) improves the blood rheology index and reduces toxicity and its incidence during radiotherapy [28]. Chaihu-huaji decoction is synthesized from small bupleurum decoction and abdominal mass pill, and in this retrospective study, we aimed to clarify the efficacy of Chaihu-huaji decoction combined with TACE for the treatment of patients with unresectable HCC, and whether the Chaihu-huaji decoction can relieve PES following TACE.

## 2. Methods

### 2.1. Patients

Patients with histologically or cytologically documented or radiographically diagnosed HCC treated in Chongqing Cancer Hospital between January 2012 and December 2014 were retrospectively reviewed. We enrolled a total of 125 patients with unresectable HCC who had received TACE treatment, of whom 64 also received Chaihu-huaji decoction (Chaihu-huaji group) and 61 received only TACE (control group). The eligibility criteria were (1) age of 18–70 years; (2) Karnofsky performance score (KPS) ≥70; (3) compensated liver function (Child-Pugh class of A or B); (4) no refractory ascites or renal failure; (5) no indication for radical operation; (6) no portal vein thrombosis or extrahepatic metastasis; and (7) no history of previous anticancer therapy.

The study was approved by our institutional review board. All patients signed an informed consent form.

### 2.2. Treatment

#### 2.2.1. TACE Procedure

TACE was performed in all patients using the Seldinger technique. After the tumor-feeding artery was identified and the catheterized hepatic arteriography was performed, chemotherapeutic agents (5-fluoruracil, 1000 mg/m^2^ and cisplatin, 80 mg/m^2^) were slowly injected into the selected feeding artery, and 5–30 mL of lipiodol with mitomycin-C (6 mg/m^2^) emulsion followed for embolization. Occasionally, a gelatin sponge was used to enhance the embolic effect. TACE was usually performed about every 4 weeks, and each session of TACE required hospitalization for 7–10 days. The treatment outcome was assessed by enhanced computed tomography (CT) within 2 weeks after TACE. The termination of treatment was based on two criteria: insufficient liver function after treatment or no residual liver tissue detected on follow-up imaging.

#### 2.2.2. CHM Administration

Patients in the Chaihu-huaji group received 200 mL of the Chaihu-huaji decoction orally twice daily. The herbal decoction was initially administered on the day of the TACE performance and was continued for 7 days after TACE. The composition of the Chaihu-huaji decoction is shown in Table 1.

### 2.3. Outcome Measurements and Follow-Up

After the last TACE session, patients were assessed every 3 months for the initial 2 years and every 6 months thereafter by enhanced CT, ultrasonography, serum biochemical analysis, and clinical examination. The patients were followed up until the last follow-up or death. The overall survival was calculated from the date of the treatment initiation to death or the date of the last follow-up.

Complications including fever, nausea, vomiting, loss of appetite, abdominal pain, and leucopenia were retrospectively analyzed in the two groups during their hospitalization. Hepatotoxicity and nephrotoxicity were assessed based on the medical records of the enrolled patients over the course until 3 months after treatment, with the use of the version 5.0 of the Common Terminology Criteria for Adverse Events (CTCAEV5.0) [29].

### 2.4. Statistical Analysis

Baseline comparisons between the two groups were performed using Student's* t *test for continuous variables and the chi-square test for categorical variables. Overall survival was analyzed by the Kaplan–Meier method, and survival curves were compared by the log-rank test. The statistical analyses were performed using adjusted chi-square test for the two groups. The Fisher's exact chi-square test was also used if the individual cell size was less than five counts. Values of* p*<0.05 were considered statistically significant. All analyses were performed with SPSS version 20.0 (IBM SPSS Inc., Chicago, IL, USA).

## 3. Results

### 3.1. General Characteristics

The patients' demographic characteristics are summarized in Table 2. No statistically significant differences in age, gender, number of tumors, main tumor size, Child-Pugh class, KPS, background liver disease, liver cirrhosis, alpha-fetoprotein (AFP) level, and Barcelona Clinic Liver Cancer stage were observed between the two groups.

### 3.2. Complications following TACE

No cases of treatment-related death were observed. Compared with the control group, fewer patients in the Chaihu-huaji group suffered from fever (10/54 versus 19/42,* p*=0.04), nausea (6/58 versus 14/47,* p*=0.038), vomiting (5/59 versus 13/48,* p*=0.032), poor appetite (7/57 versus 17/44,* p*=0.015), and leukocytopenia (3/61 versus 11/50,* p*=0.018). Most patients developed pain (29 in the Chaihu-huaji group versus 34 in the control group,* p*=0.224) after TACE. Five patients in the Chaihu-huaji group and eight in the control group (*p*=0.332) experienced moderate intraperitoneal hemorrhage. The observed complications are summarized in Table 3.

### 3.3. Drug-Related Adverse Events

No cases of significant hepatic and renal toxicities related to the herbal medicine were observed in the Chaihu-huaji group (Table 4). Liver failure (Child-Pugh class C) occurred in 21 cases in the Chaihu-huaji group and 15 cases in the control group (*p*=0.446), and 13 patients in the Chaihu-huaji group and 10 in the control group developed abnormal serum creatinine (Cr,* p*=0.572).

### 3.4. Overall Survival

**Figure 1:**
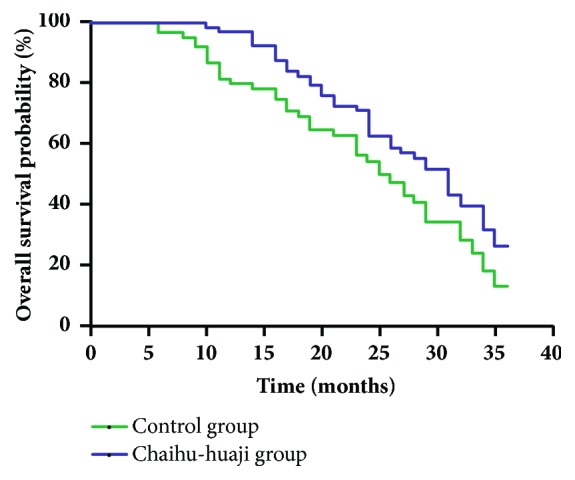


During the first 3 years after treatment initiation, 40 of 64 patients in the Chaihu-huaji group and 42 of 61 patients in the control group died. Among them, 19 in the Chaihu-huaji group and 27 in the control group died of tumor progression, while other deaths were caused by hepatic failure. The 3-year overall survival was significantly higher in the Chaihu-huaji group than in the control group (26.47% versus 13.06%; hazard ratio [HR]=1.607; 95% confidence interval [CI]=1.020–2.533;* p*=0.0408 by log-rank test; Figure 1).

## 4. Discussion

TACE is recommended as the first-line treatment for patients with unresectable and/or large/multifocal HCC, with no vascular invasion or extrahepatic spread, and with satisfactory liver function (Child-Pugh A or B); this treatment should be repeated at intervals of 2–3 months based on the assessment of tumor status and liver function [30]. A low treatment success rate is mainly caused by adverse events, most of which are related to PES, including liver enzyme abnormalities (18.1%), fever (17.2%), abdominal pain (11.0%), vomiting (6.0%), and nausea (1.7%), and hematological/bone marrow toxicity is estimated to occur in 13.5% of patients [31, 32].

In traditional Chinese medicine (TCM), chemotherapeutics and embolic agents are considered as the exotic pathogenic qi for damage to the liver and spleen and struggles with healthy qi. Pathogenic qi is between the exterior and interior. The symptoms include alternating fever and chills; fullness and discomfort in the chest and hypochondrium; anorexia, vexation, and vomiting; bitter taste and dry pharynx; and dizziness, thin white tongue fur, and wiry pulse. Embolization of the tumor blood supply during TACE can depress qi, block venation, and aggravate the degree of qi stagnation and blood stasis. Therefore, the symptom of abdominal pain appears.

Previous clinical studies have demonstrated the utility of TCM in relieving adverse events after TACE. Tang et al. [16] found that CHM was effective at reducing side effects and improving long-term survival in cases of unresectable HCC treated with TACE. Xu et al. [21] found that Jian Pi Li Qi (JPLQ) decoction can relieve PES (including fever, pain, fatigue, and lack of appetite) and improve the QOL of patients after TACE. Our study has shown that Chaihu-huaji decoction can relieve the symptoms of fever, nausea, vomiting, poor appetite, and leukocytopenia. Liver and kidney functional damage may be result from the TACE procedure, and CHM might aggravate these insults. However, the present study did not show statistically significant differences in liver and kidney function between the group treated with CHM and that treated without CHM.

Small bupleurum decoction and abdominal mass pill are widely used in China. The Chaihu-huaji decoction used in this study is derived from the integration of Xiao Chaihu decoction and Hua Ji pill. It functions to sooth the liver, invigorate the spleen, eliminate pathogenic factors, and move qi to dissipate blood stasis. Xiao Chaihu decoction originated from the treatise of Cold Damage Diseases written by Zhang Zhongjing at the end of the Eastern Han Dynasty. Hua Ji pill is derived from the Rhinoceros Candle of the Source of the Disease. Bupleurum root and Scutellaria root, bitter and cold in properties, are the core medicinal pair of the decoction.* Pinellia ternata*, a pungent-warm herb, can invigorate the spleen and harmonize the stomach. When combined with ginger, these herbs, as the antiemetic, dissipate adversely rising qi. Ginseng and* Glycyrrhiza uralensis* tonify the healthy qi to prevent the return of the pathogenic qi.* Rhizoma sparganii* trigmae,* Rhizoma Zedoary*, and hematoxylin promote blood circulation and prevent blood stasis. Cyperus, areca, and* Fructus Aurantii* regulate qi and relieve pain. Bryozoatum and* Concha Arcae* eliminate stagnation. This TCM formula can eliminate pathogenic qi and maintain healthy qi, restore the functions of the liver and spleen, and make qi moving smoothly and blood unblocked.

Recently, CHM is emerging as an intriguing and viable choice because of its multilevel, multitarget, and coordinated intervention effects against HCC [33]. Although further research is needed to elucidate the detailed mechanisms of the CHM-mediated anticancer effects, evidence that has accumulated in the past several decades confirms the preventive and therapeutic effects of CHM against HCC. A recent randomized controlled study showed that CHM prevents postoperative recurrence of small HCC and prolongs overall survival [34]. In the present study, the Chaihu-huaji decoction group had an improved 3-year overall survival.

More cellular and molecular mechanisms of the anti-HCC activity of various CHMs have been uncovered [33]. For instance, baicalein, a flavonoid in the Chaihu-huaji decoction, exerts anti-HCC effects by inhibiting the activity of topoisomerase II and suppressing the proliferation of HCC cell lines in vitro [35]; it acts on broad cell signaling networks and leads to a collective inhibition of cell proliferation [36]. The decoction has proven powerful in regulating HCC invasion and metastasis [37].

## 5. Conclusion

In summary, the present results demonstrate that Chaihu-huaji decoction may reduce the occurrence of side effects after TACE and improve the overall survival of patients with unresectable HCC when combined with TACE.

## Figures and Tables

**Table 1:** Herbal composition of Chaihu-huaji decoction.

Name of herb	Amount (g)
*Radix bupleuri*	30
*Scutellaria baicalensis*	9
*Pinellia ternata*	9
Ginseng	9
*Rhizoma sparganii*	10
*Curcuma zedoary*	10
Hematoxylon	6
Fresh ginger	9
*Rhizoma cyperi*	10
Betel nut	6
*Fructus aurantii*	15
Bryozoatum	30
*Concha arcae*	30

**Table 2:** Patient characteristics.

Characteristic	Chaihu-huaji group	Control group	*p*
	(n=64)	(n=61)	
Age (years)	47.51 ± 10.32	46.36 ± 9.67	0.52
Gender			
Male	52	51	0.729
Female	12	10	
Number of tumors			
1	14	20	0.363
2	19	12	
3	19	15	
	12	14	
Main tumor size (cm)	6.20 ± 0.85	6.27 ± 0.87	0.648
Child–Pugh class			
A	45	40	0.57
B	19	21	
KPS			
70	15	9	0.572
80	26	24	
90	18	22	
100	5	6	
Background liver disease			
HBV infection	48	51	0.411
HCV infection	10	5	
Others	6	5	
Liver cirrhosis	52	52	0.55
AFP level (ng/mL)			
	11	8	0.354
100-400	18	12	
	35	41	
Barcelona Clinic Liver Cancer stage			
I	7	6	0.733
II	21	23	
III	36	32	

KPS, Karnofsky performance score; HBV, hepatitis B virus; HCV, hepatitis C virus; AFP, alpha-fetoprotein.

**Table 3:** Complications following TACE.

Complication	Chaihu-huaji group	Control group	*p*
	(n=64)	(n=61)	
Fever (n)	10	19	0.04
Pain (n)	29	34	0.224
Nausea (n)	6	14	0.038
Vomiting (n)	5	13	0.032
Poor appetite (n)	7	17	0.015
Hemorrhage (n)	5	8	0.332
Leukocytopenia (n)	3	11	0.018

TACE, transarterial chemoembolization.

**Table 4:** Drug-related adverse events.

Adverse event	Chaihu-huaji group	Control group	*p*
	(n=64)	(n=61)	
Child-Pugh class C (n)	21	15	0.31
Abnormal serum Cr (n)	13	10	0.572

Cr, creatinine; abnormal serum Cr: exceeding 1.5 times the upper limit of normal Cr.

## Data Availability

The data used to support the findings of this study are available from the corresponding author upon request.
